# Pituitary adenylate cyclase-activating peptide induces long-lasting neuroprotection through the induction of activity-dependent signaling via the cyclic AMP response element-binding protein-regulated transcription co-activator 1

**DOI:** 10.1111/j.1471-4159.2011.07330.x

**Published:** 2011-08

**Authors:** Paul S Baxter, Marc-Andre Martel, Aoife McMahon, Peter C Kind, Giles E Hardingham

**Affiliations:** Centre for Integrative Physiology, University of EdinburghEdinburgh, UK

**Keywords:** Ca^2+^ signaling, CREB, gene regulation, neuroprotective signalling, neurotoxicity, transcription factors

## Abstract

Pituitary adenylate cyclase-activating peptide (PACAP) is a neuroprotective peptide which exerts its effects mainly through the cAMP-protein kinase A (PKA) pathway. Here, we show that in cortical neurons, PACAP-induced PKA signaling exerts a major part of its neuroprotective effects indirectly, by triggering action potential (AP) firing. Treatment of cortical neurons with PACAP induces a rapid and sustained PKA-dependent increase in AP firing and associated intracellular Ca^2+^ transients, which are essential for the anti-apoptotic actions of PACAP. Transient exposure to PACAP induces long-lasting neuroprotection in the face of apoptotic insults which is reliant on AP firing and the activation of cAMP response element (CRE) binding protein (CREB)-mediated gene expression. Although direct, activity-independent PKA signaling is sufficient to trigger phosphorylation on CREB’s activating serine-133 site, this is insufficient for activation of CREB-mediated gene expression. Full activation is dependent on CREB-regulated transcription co-activator 1 (CRTC1), whose PACAP-induced nuclear import is dependent on firing activity-dependent calcineurin signaling. Over-expression of CRTC1 is sufficient to rescue PACAP-induced CRE-mediated gene expression in the face of activity-blockade, while dominant negative CRTC1 interferes with PACAP-induced, CREB-mediated neuroprotection. Thus, the enhancement of AP firing may play a significant role in the neuroprotective actions of PACAP and other adenylate cyclase-coupled ligands.

Pituitary adenylate cyclase-activating peptide (PACAP) is a neuropeptide first isolated from the hypothalamus as an activator of cAMP production in pituitary cells (Miyata *et al.* 1989). It exists in 27 and 38-amino acid forms and binds to three G-protein coupled receptors [PACAP-specific receptor (PAC1) and VIP/PACAP receptor subtypes 1 and 2] which are predominantly coupled to Gαs that promote cAMP production through the activation of adenylate cyclase (AC) (Dickson and Finlayson 2009). PACAP and its receptors are expressed widely in the CNS, where one of their key functions is neuroprotection. PACAP promotes the protection of cerebellar granule neurons against apoptotic and oxidative insults including ceramide, ethanol and H_2_O_2_ (Vaudry *et al.* 2009). PACAP also protects cortical and hippocampal neurons against excitotoxic and apoptotic insults (Shioda *et al.* 1998; Vaudry *et al.* 2009). *In vivo*, administration of PACAP reduces neuronal loss and neurological deficits in models of stroke and traumatic brain injury (Reglodi *et al.* 2002; Chen *et al.* 2006; Tamas *et al.* 2006b; Vaudry *et al.* 2009), excitotoxic striatal lesions (Tamas *et al.* 2006a) and Parkinson’s disease (Reglodi *et al.* 2004, 2006). Given this, PACAP has received considerable attention as a potential therapeutic neuroprotective drug (Somogyvari-Vigh and Reglodi 2004; Shioda *et al.* 2006; Brenneman 2007; Ohtaki *et al.* 2008; Vaudry *et al.* 2009).

PACAP promotes neuroprotection by acting directly on neuronal PACAP receptors (Vaudry *et al.* 2009). The molecular mechanisms that underlie this neuroprotection centre on activation of the cAMP-dependent protein kinase A (PKA), a major effector of intracellular cAMP (Botia *et al.* 2007; Vaudry *et al.* 2009). Activation of *de novo* gene expression has been implicated in PACAP-mediated neuroprotection, including c-Fos, brain-derived neurotrophic factor, Bcl-2 and PACAP itself (Frechilla *et al.* 2001; Falluel-Morel *et al.* 2004; Shintani *et al.* 2005; Aubert *et al.* 2006; Dejda *et al.* 2008). Of note, these genes are all regulated by the cAMP response element (CRE) binding protein (CREB) family of transcription factors, a group of factors that are important for the survival of central and peripheral neurons both pre- and postnatally (Walton *et al.* 1999; Lonze *et al.* 2002; Mantamadiotis *et al.* 2002) and whose activation contribute to the neuroprotective effects of neurotrophins and synaptic activity (Bonni *et al.* 1999; Riccio *et al.* 1999; Lee *et al.* 2005; Papadia *et al.* 2005). PACAP is known to promote CREB activation under conditions where it is neuroprotective (Racz *et al.* 2006; Falktoft *et al.* 2009), however, a causal link has up until now not been tested.

It is generally assumed that PACAP-mediated PKA signaling in neurons triggers neuroprotective gene expression and signal pathways by direct modulation of upstream effectors of these processes. However, we have considered an alternative explanation: that PACAP-induced PKA signaling exerts at least some of its neuroprotective effects indirectly though the enhancement of electrical activity. G-protein coupled receptors that activate cAMP/PKA signals in neurons, such as type I mGluRs and D_1_-type dopamine receptors, can potentiate synaptic strength and neuronal excitability, and modulate ion channel properties (Nguyen and Woo 2003). PACAP administration has been recently reported to enhance AMPAR currents as well as synaptic NMDAR currents (MacDonald *et al.* 2007; Costa *et al.* 2009) and to suppress the Apamin-insensitive slow after-hyperpolarization (IsAHP) current (Hu *et al.* 2011), which can control neuronal excitability.

Physiological patterns of action potential (AP) bursting are known to be strongly neuroprotective (Bell and Hardingham 2011), activating multiple pathways including CREB-mediated gene expression, antioxidant gene expression and the suppression of apoptotic genes (Hardingham 2006; Hetman and Kharebava 2006; Al-Mubarak *et al.* 2009; Hardingham and Bading 2010; Soriano *et al.* 2011; Zhang *et al.* 2011). An episode of burst activity can confer neuroprotection long after that episode has ceased, via a mechanism involving the activation of nuclear Ca^2+^- and CREB-dependent gene expression (Papadia *et al.* 2005; Hardingham 2009; Zhang *et al.* 2009). Thus, we have studied the effect of PACAP on levels of electrical activity in cortical neurons, and the role this plays in neuroprotection. We find that PACAP-induced PKA signaling triggers sustained increases in AP firing and that this firing activity is essential for PACAP-mediated neuroprotection. Specifically, PACAP-induced AP firing is required in order to trigger nuclear translocation of CREB-regulated transcription co-activator 1 (CRTC1, previously referred to as TORC1: Transducer Of Regulated CREB activity 1) in order to activate CREB-mediated gene expression and subsequent neuroprotection.

## Materials and methods

### Neuronal cultures and chemicals used

Cortical neurons from E21 Sprague–Dawley rats were cultured as described (Bading and Greenberg 1991; McKenzie *et al.* 2005) except that growth medium was comprised of Neurobasal A medium with B27 (Invitrogen, Carlsbad, CA, USA), 1% rat serum (Harlan Inc., Indianapolis, IN, USA), 1 mM glutamine. Experiments were performed after a culture period of 9–10 days during which neurons developed a rich network of processes, expressed functional NMDA-type and α-amino-3-hydroxy-5-methylisoxazole-4-propionate (AMPA)/kainate-type glutamate receptors, and formed synaptic contacts (Hardingham *et al.* 2001, 2002). PKA RIIβ wild-type and knockout mice (Brandon *et al.* 1998; Watson *et al.* 2006) were cultured as above from E17 animals. PACAP-27 was purchased from NeoMPS (Strasbourg, France). PACAP-27 and PACAP-38 are both found in the brain, and PACAP-27 was chosen since it represents the core functional region for activating PACAP receptors (Vaudry *et al.* 2009). Since we were concerned with events *downstream* of PACAP receptor activation, PACAP-27 was deemed sufficient for this purpose. MK801, KN-62 and forskolin from Tocris Cookson, Ballwin, MO, USA, bicuculline from Sigma, St Louis, MO, USA, PD-98059 from Ascent Scientific (Bristol, UK), H-89 from LC Laboratories (Woburn, MA, USA), staurosporine, tetrodotoxin (TTX) and 4-aminopyridine from Calbiochem, San Diego, CA, USA.

### Models of neuronal apoptosis, PACAP-induced protection

#### Trophic deprivation

Neurons were transferred from growth medium to a trophically-deprived medium containing 10% minimal essential medium (Invitrogen), 90% Salt-Glucose-Glycine (SGG) medium (Papadia *et al.* 2005) (SGG: 114 mM NaCl, 0.219% NaHCO_3_, 5.292 mM KCl, 1 mM MgCl_2_, 2 mM CaCl_2_, 10 mM HEPES, 1 mM glycine, 30 mM glucose, 0.5 mM sodium pyruvate, 0.1% Phenol Red; osmolarity 325 mOsm/L). When placed in this trophically-deprived medium, neurons exhibit significant levels of caspase-dependent apoptosis after 72 h (Papadia *et al.* 2005; Leveille *et al.* 2010). To assess neuroprotective signaling by continuous PACAP exposure, PACAP-27 (10 nM, NeoMPS) was administered at the point of trophic deprivation and left throughout the course of the experiment (72 h). The importance of PACAP-induced firing activity for neuroprotection was assessed by administering PACAP in the presence or absence of tetrodotoxin (1 μM). To assess the long-lasting neuroprotective effect of PACAP exposure (and its dependence on firing activity), PACAP ± TTX was administered at the point of trophic deprivation for 24 h, after which the medium was replaced with PACAP-free, TTX-containing medium. Cell death was quantified after a total of 72 h trophic deprivation.

#### Staurosporine-induced apoptosis

To induce rapid apoptotic cell death, and assess PACAP-induced protection, staurosporine treatment was employed as described (Papadia *et al.* 2005; Leveille *et al.* 2010). Briefly, neurons were placed in trophically deprived medium ± PACAP for 23 h, at which point the PACAP-treated neurons were given a second dose of PACAP. After a further 1 h, neurons were treated with 100 nM staurosporine, and death was assessed after a further 24 h. We have previously established that 100 nM staurosporine induced caspase-dependent apoptosis over this time period (Papadia *et al.* 2005; Leveille *et al.* 2010).

### Assessment of cell death

To quantify cell death, neurons were fixed and subjected to DAPI (Vector Laboratories, Burlingame, CA, USA) staining and cell death quantified by counting (blind) the number of apoptotic nuclei as a percentage of the total. Approximately 1500 cells were counted per treatment, across at least four independent experiments (performed on different cultures). Morphologically, staurosporine-treated and trophically deprived neurons show typical signs of apoptotic-like cell death (shrunken cell body and large round chromatin clumps). Images were taken using a Leica AF6000 LX imaging system (Milton Keynes, UK), with a DFC350 FX digital camera.

### Calcium imaging and analysis of imaging data

For pre-conditioning experiments, neurons were treated as indicated for 2 h, then transferred to aCSF (150 mM NaCl, 3 mM KCl, 10 mM HEPES, 2 mM CaCl_2_, 1 mM MgCl_2_, 1 mM glucose) Ca^2+^ imaging was performed as described (Hardingham *et al.* 1997; Soriano *et al.* 2008b). Briefly, cells were loaded with 11 μM Fluo-3 AM [from a stock solution of 2.2 mM Fluo-3 dissolved in anhydrous dimethylsulfoxide containing 20% (w/v) Pluronic detergent] for 30 min at 37°C. Fluo-3 fluorescence images (excitation 488 nm, emission ≥ 515 nm) were taken at one frame per second. To calibrate images, Fluo-3 was saturated by adding 50 μM ionomycin to the perfusion chamber (to obtain *F*_max_) and quenched with 10 mM MnCl_2_ +50 μM ionomycin to levels corresponding to 100 nM Ca^2+^ (Minta *et al.* 1989), which was in turn used to calculate *F*_min_. Free Ca^2+^ concentrations were calculated from fluorescence signal (*F*) according to the equation [Ca^2+^] = *K*_d_(*F*–*F*_min_)/(*F*_max_–*F*), and expressed as a multiple of the *K*_d_ of Fluo-3 (which is approximately 315 nM). In order to quantitate the effect of PACAP on firing activity-induced Ca^2+^ influx, the mean [Ca^2+^] 30 s before and 30 s after TTX treatment was calculated in either control neurons or neurons treated with PACAP ± H-89. For each cell, the degree of TTX-sensitive Ca^2+^ changes was calculated as the difference between mean [Ca^2+^] before and after TTX treatment. For each treatment, 60 cells were analysed within six independent experiments.

### Electrophysiological recording and analysis

Coverslips containing cortical neurons were transferred to a recording chamber and perfused (at a flow rate of approximately 5 mL/min) with an external recording solution composed of 150 mM NaCl, 2.8 mM KCl, 10 mM HEPES, 2 mM CaCl_2_, 1 mM MgCl_2_, 50 μM glycine, 2 μM strychnine and 10 mM glucose, pH 7.3 (320–330 mOsm). Patch-pipettes were made from thick-walled borosilicate glass (Harvard Apparatus, Kent, UK) and filled with a K-gluconate-based internal solution containing (in mM): 155 K-gluconate, 2 MgCl_2_, 10 Na-HEPES, 10 Na-PiCreatine, 2 Mg_2_-ATP and 0.3 Na_3_-GTP, pH 7.3 (300 mOsm). Electrode tips were fire-polished for a final resistance ranging between 5 and 10 MΩ. Currents were recorded at room temperature (21 ± 2°C) using an Axopatch-1C amplifier (Molecular Device, Union City, CA, USA) and stored on digital audio tape. Data were subsequently digitized and analyzed using WinEDR v6.1 software (John Dempster, University of Strathclyde, UK). Neurons were voltage-clamped at −70 mV, and recordings were rejected if the holding current was greater than −100 pA or if the series resistance drifted by more than 20% of its initial value (< 35 MΩ). Neurons were treated ± PACAP (10 nM) for 1–2 h prior to spontaneous excitatory post-synaptic currents being recorded in voltage-clamp for 5 min. Recordings were studied to determine whether they showed evidence of burst-like activity, defined as long periods of activity (> 1 s), peaking at > 50 pA.

### Western blotting

In order to minimize the chance of post-translational modifications during the harvesting process, neurons were lysed immediately in 1.5× sample buffer (1.5 M Tris pH 6.8; Glycerol 15%; sodium dodecyl sulfate 3%; β-mercaptoethanol 7.5%; bromophenol blue 0.0375%) and boiled at 100°C for 5 min. Approximately 30 μg of protein was loaded onto a gel and subjected to gel electrophoresis and western blotting were performed using the Xcell Surelock system (Invitrogen) with precast gradient gels (4–20%) according to the manufacturer’s instructions. The gels were blotted onto polyvinylidene difluoride membranes, which were blocked for 1 h at 21 ± 2°C with 5% (w/v) non-fat dried milk in Tris-buffered saline with 0.1% Tween 20. The membranes were incubated at 4°C overnight with the primary antibodies diluted in blocking solution: Anti-phospho-CREB serine-133 (1 : 500, Upstate Biotechnology, Lake Placid, NY, USA) and CREB (1 : 500, Upstate). For visualisation of western blots, horseradish peroxidase-based secondary antibodies were used followed by chemiluminescent detection on X-Omat film (Kodak, Hemel Hempstead, UK). Western blots were analyzed by digitally scanning the blots, followed by densitometric analysis (ImageJ, National Institutes of Health, Washington DC, USA). All analysis involved normalizing to CREB expression as a loading control.

### Transfection and luciferase assays

Neurons were transfected at DIV8 using Lipofectamine 2000 as described (McKenzie *et al.* 2006) using a total of 0.6–0.7 μg cDNA/well and 2.33 μL/well of Lipofectamine 2000 (1 μg/mL, from Invitrogen). Under these conditions transfection efficiency is approximately 2–5%, with > 99% of transfected cells NeuN-positive, and < 1% glial fibrillary acidic protein-positive (Papadia *et al.* 2008; Soriano *et al.* 2008a) confirming their neuronal identity. For CRE-reporter assays, neurons were transfected with 0.5 μg of CRE-Firefly Luciferase + 0.1 μg of pTK-Renilla (Promega, Madison, WI, USA); or 0.2 μg CRE-Luc, 0.1 μg Renilla and 0.4 μg of either β-globin control vector, or vectors encoding inducible cAMP early repressor 1 (ICER1) [a gift from Dr. Paulo Sassone-Corsi (Stehle *et al.* 1993)], CRTC1 (TORC1) or Dominant negative CRTC1 [CRTC1-DN (TORC1-N44), a gift from Dr Yang Zhou (Zhou *et al.* 2006)]. Stimulations were performed 24 h post-transfection. Neurons were treated with 10 nM PACAP or 5 μM Forskolin for 4 h, or with 50 μM bicuculline and 250 μM 4-aminopyridine for 8 h, with inhibitors added 1 h before. Assays were performed using the Dual Glo assay kit (Promega) and were performed on a FLUOstar OPTIMA (BMG Labtech, Aylesbury, UK). Firefly luciferase activity was normalized to the Renilla control in all cases and each experiment was performed at least four times.

### Following the fate of transfected cells

The overall method to do this is as described (Papadia *et al.* 2008) with some modifications to the timing. Neurons were transfected with 0.5 μg of vectors expressing β-globin or ICER1 or CRTC1-DN plus 0.1 μg of a plasmid encoding enhanced green fluorescent protein (GFP) as a transfection marker. At 24 h post-transfection, neurons were placed in trophically-deprived medium and treated ± 10 nM PACAP. After a further 24 h, images were taken of GFP-expressing neurons using a Leica AF6000 LX imaging system, with a DFC350 FX digital camera, prior to the transfer of cell to PACAP-free, TTX-containing medium to block AP firing. Using mark-and-find software, the fate of the photographed neurons was followed at 24 h and 48 h after TTX treatment. 250–500 cells were analysed per treatment within 3–5 independent experiments.

### CRTC1-localisation

For CRTC1-localisation studies, neurons were transfected with 0.6 μg GFP tagged CRTC1 [peGFP-C2/TORC1 a gift from Dr Dong-Yan Jin, Department of Biochemistry, University of Hong Kong, Hong Kong China (Siu *et al.* 2006)]. At 24 h post-transfection, neurons were treated with 20 ng/mL Leptomycin B (LC Laboratories) for 30 min in order to visualise CRTC1 import more clearly (Kovacs *et al.* 2007) and then 10 nM PACAP for a further 30 min in the presence of 1 μM TTX, 10 μM H-89 or 10 μM FK-506 (added 1 h before). Neurons were then fixed and stained for anti-GFP (1 : 750, Invitrogen) and visualised using biotinylated secondary antibody/cy3-conjugated streptavidin. Nuclei were counter-stained with DAPI. Subcellular distribution of CRTC1 was scored as being nuclear if levels were higher in the nucleus than in the surrounding perinuclear cytoplasm. 400–800 cells were analysed per treatment across 4–8 independent experiments.

### Statistical analysis

Statistical testing involved a 2-tailed paired Student’s *T*-test. For studies employing multiple testing, we used a one-way ANOVA followed by Fisher’s least significant difference *post-hoc* test.

## Results

### PACAP triggers sustained increases in AP firing in cortical neurons

**Fig. 1:**
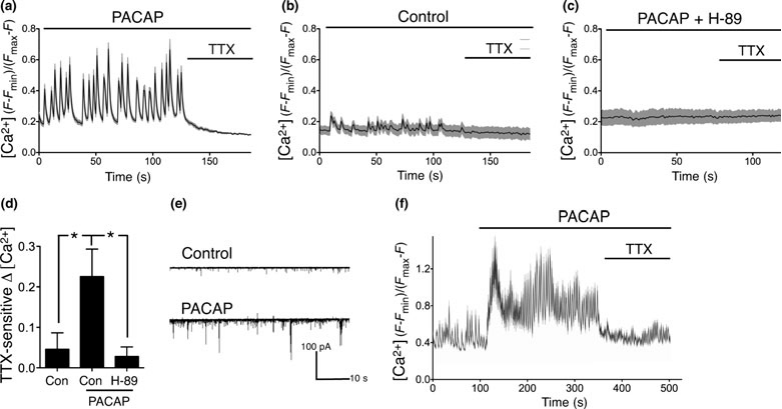
PACAP enhances AP firing in cortical neurons. (a–d) Pre-treatment with PACAP (10 nM PACAP-27 here and throughout the study) causes an increase in AP firing-dependent Ca^2+^ transients. Neurons were treated where indicated with PACAP ± H-89 (10 μM). After 2 h, the neurons were subjected to Fluo-3 Ca^2+^ imaging studies (see Methods for details) to monitor the size of Ca^2+^ transients in the different stimulation conditions. TTX (1 μM) was added where indicated to determine the extent to which the observed Ca^2+^ transients were because of action potential firing. Example traces are shown: black line indicates the mean Ca^2+^ concentration within a field of cells, and the grey shaded region indicates ± SEM of the Ca^2+^ concentration within that field. Free Ca^2+^ concentrations were calculated from fluorescence signal (*F*) according to the equation [Ca^2+^] = *K*_d_(*F*–*F*_min_)/(*F*_max_–*F*), and expressed as a multiple of the *K*_d_ of Fluo-3 (which is approximately 315 nM). (d) shows quantification of data shown in (a–c), that is, quantification of the difference in mean amplitude of [Ca^2+^] before and after 1 μM TTX treatment. In order to quantitate the effect of PACAP on firing activity-induced [Ca^2+^] influx, the mean [Ca^2+^] 30 s before and 30 s after TTX treatment was calculated in either control neurons or neurons treated with PACAP ± H-89. For each cell, the degree of TTX-sensitive Ca^2+^ changes was calculated as the difference between mean [Ca^2+^] before and after TTX treatment. For each condition, 60 cells were analysed within six independent experiments (*p < 0.05). (e) Example trace of a whole-cell voltage-clamp recording of a control and PACAP-treated cortical neurons. PACAP causes an increase in burst-like activity, consistent with the Ca^2+^ imaging data. (f) Ca^2+^ imaging of acute PACAP treatment, a typical example trace is shown representative of six independent experiments.

PACAP is known to promote PKA-dependent neuroprotection in a variety of systems *in vitro* and *in vivo* (see above). However, PKA activation is also capable of altering neuronal network activity through the control of intrinsic excitability and synaptic strength (Nguyen and Woo 2003). To investigate the effect of PACAP on levels of electrical activity we performed Ca^2+^ imaging experiments on cortical neurons pre-treated with PACAP. This pre-treatment resulted in enhanced AP firing as evidenced by strong oscillatory intracellular Ca^2+^ transients that were blocked by the Na^+^ channel antagonist TTX (Fig. 1a, quantitation in Fig. 1d). In contrast, control neurons exhibited far smaller TTX-sensitive Ca^2+^ transients (Fig. 1b and d). Pre-treatment of neurons with the PKA inhibitor H-89 prevented any PACAP-induced changes in Ca^2+^ oscillations (Fig. 1c and d). We also performed whole-cell voltage-clamp recordings of neurons pre-treated with PACAP which corroborated the Ca^2+^ imaging data: nine out of nine PACAP-treated neurons exhibited incoming excitatory post-synaptic currents consistent with burst-like activity (> 1 s in duration, > 50 pA at peak), compared to zero out of eight control neurons (Fig. 1e). Further Ca^2+^ imaging experiments revealed that acute administration of PACAP also had a similar effect, indicating that the potentiating effect of PACAP on AP firing is fast-acting as well as long-lasting (Fig. 1f). Thus, PACAP-induced PKA signaling in cortical neurons induces long-lasting increases in AP firing and associated Ca^2+^ transients.

### Enhanced AP firing is essential for PACAP-induced neuroprotection

**Fig. 2:**
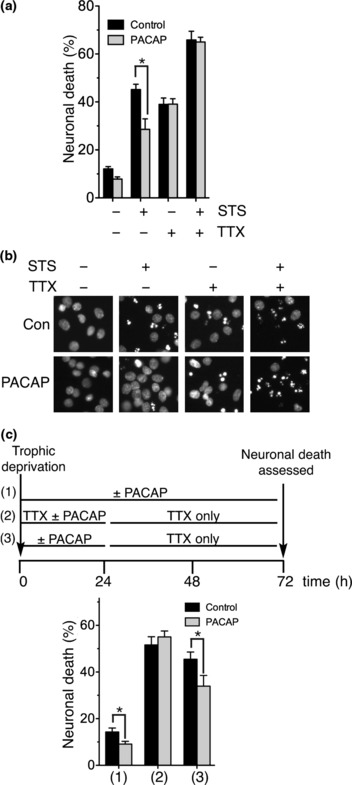
PACAP promotes resistance to apoptotic stimuli which is dependent on AP firing. (a,b) PACAP protects against staurosporine-induced cell death, but not in the presence of TTX. Neurons were treated with PACAP in the presence or absence of TTX 24 h and 1 h before treatment with 100 nM staurosporine. After a further 24 h, the cells were then fixed and DAPI stained and death was measured by counting pyknotic and non-pyknotic nuclei (*p < 0.05, *n* = 4); (b) shows example pictures. (c) PACAP-induced AP firing protects against trophic deprivation and promotes long lasting neuroprotection. At *t* = 0, the neurons were placed in trophically-deprived medium and given one of the three treatment regimes outlined in the upper schematic (1–3). At *t* = 72 h, cells were fixed, DAPI stained and levels of neuronal death analysed (*p < 0.05, *n* = 3).

It is known that elevated electrical activity can promote neuroprotection in cortical neurons (Mennerick and Zorumski 2000; Bell and Hardingham 2011), raising the possibility that PACAP-induced AP firing contributes to its neuroprotective effect. We studied the capacity of PACAP to protect neurons against two different apoptotic insults, and studied the effect of blocking AP firing by TTX treatment. We first used staurosporine which induces caspase-dependent apoptosis of cortical neurons (Papadia *et al.* 2005). Pre-treatment of cortical neurons with PACAP before exposure to staurosporine for 24 h reduced levels of apoptosis (Fig. 2a and b). TTX treatment alone enhanced basal levels of neuronal apoptosis, however, staurosporine treatment caused additional neuronal loss. Importantly, we found that PACAP treatment failed to protect neurons against apoptosis in the presence of TTX.

We next employed a model of prolonged trophic deprivation (72 h) that also induces progressive caspase-dependent apoptosis (Papadia *et al.* 2005; Leveille *et al.* 2010). Once again, PACAP treatment protected neurons against apoptosis in control, although overall levels of apoptosis were not high [Fig. 2c, treatment (1)]. An episode of AP firing can promote neuroprotection that lasts well beyond the point at which that activity ends (Papadia *et al.* 2005). We hypothesised that PACAP-induced AP firing would similarly be able to exert long-lasting neuroprotection. Neurons subjected to trophic deprivation were treated with or without PACAP for 24 h, after which all neurons were placed in PACAP-free, TTX-containing medium. Levels of apoptosis were then assessed after a further 48 h [Fig. 2c, treatment (3)]. PACAP treatment was found to confer significant neuroprotection [Fig. 2c, treatment (3)] and this was dependent on PACAP-induced AP firing, since no protection was observed if PACAP was administered in the presence of TTX [Fig. 2c, treatment (2)]. Thus, PACAP-induced AP firing confers long-lasting neuroprotection. We conclude from these experiments that PACAP-induced enhancement of AP firing is important for its neuroprotective effects in these models of cortical neuronal apoptosis. This suggests that activation of PKA signaling is insufficient to *directly* activate certain neuroprotective pathways, and that it activates them *indirectly* by inducing AP firing which in turn triggers Ca^2+^-dependent signaling pathways that induce pro-survival events.

### Induction of CREB-mediated gene expression contributes to PACAP-mediated neuroprotection

**Fig. 3:**
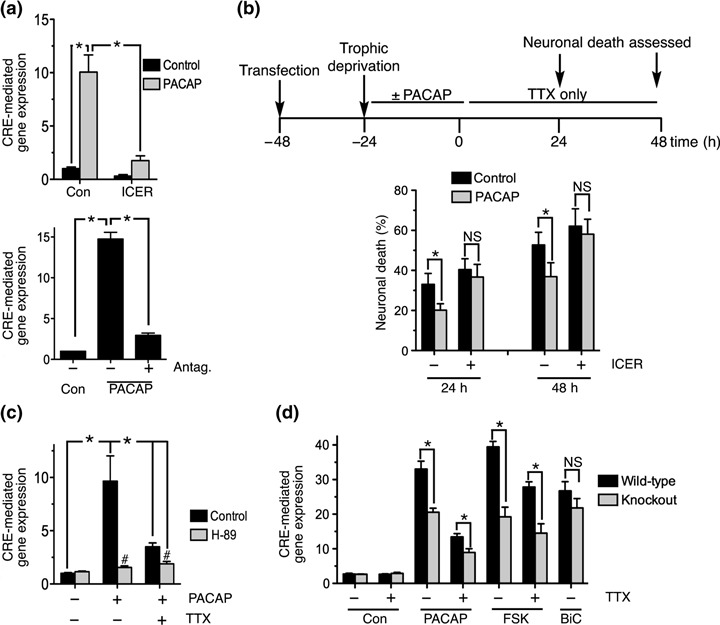
PACAP induces CRE-dependent gene expression, which is neuroprotective, and relies on AP firing. (a) *Upper***-**PACAP induces CRE-mediated gene expression. Neurons were transfected with a CRE-Firefly luciferase vector, pTK renilla transfection control and vectors encoding either ICER1 or control (β-globin). See Methods section for exact quantities used. At 24 h post-transfection, neurons were treated with PACAP and luciferase expression was measured after a further 4 h. CRE-Firefly luciferase activity was normalised to Renilla control (*p < 0.05, *n* = 3). *Lower*-Effect of the PACAP antagonist (Antag, PACAP6–38, 1 μM) on PACAP induction of CRE-luciferase (*p < 0.05, *n* = 3). (b) PACAP mediated long-lasting neuroprotection depends on activation of CRE-mediated gene expression. Upper panel illustrates the experimental protocol. Briefly, neurons expressing GFP plus either ICER1 or β-globin control were treated ± PACAP 24 h post-transfection and then all cells were placed in TTX-containing medium after a further 24 h, at which point images of GFP-expressing neurons were taken (*t* = 0 in the upper schematic). The fate of these cells was then monitored at 24 and 48 h after this medium change. 250–400 cells were analysed per treatment across six cultures within three independent experiments. (*p < 0.05). (c) PACAP induced CRE-dependent gene expression is dependent on AP firing. Neurons were treated with PACAP where indicated for 4 h; all other drugs were added 1 h beforehand (*p < 0.05, ^#^*p* < 0.05 comparing H-89 with control for that particular PACAP/TTX condition, *n* = 7). (d) PACAP and forskolin-induced activation of CRE-mediated gene expression is disrupted in RIIβ-deficient neurons: both AP firing-dependent and independent components. Forskolin was used at 5 μM. For comparison is an illustration of the RIIβ-*independence* of CRE activation triggered by promoting AP firing by network disinhibition through treatment with the GABA_A_ receptor blocker bicuculline (50 μM) plus 250 μM 4-aminopyridine, which is a PKA-independent way of inducing AP firing (Papadia *et al.* 2005) (*p < 0.05, *n* = 6).

We next investigated the mechanism by which PACAP-induced AP firing leads to long-lasting neuroprotection. The CREB family controls the expression of a number of pro-survival genes containing CRE promoter elements and is a target for activation by both cAMP/PKA signals as well as activity-dependent Ca^2+^ signaling (Lonze and Ginty 2002). CREB itself is the predominant member in forebrain neurons (Papadia *et al.* 2005) and so is likely to be responsible for the majority of CRE-mediated gene expression. PACAP treatment resulted in the strong activation of a CRE-reporter (which was blocked by a PACAP receptor antagonist, Fig. 3a, lower), raising the possibility that CREB activation contributes to PACAP-mediated neuroprotection. We studied the effect of blocking CRE-dependent gene expression by transfecting neurons with a vector encoding ICER1, which is an inhibitory isoform of the CREB family (De Cesare and Sassone-Corsi 2000). We confirmed the efficacy of ICER1: expression of ICER1 blocked PACAP-induction of a CRE-reporter gene (Fig. 3a, upper).

To assess the importance of CREB in PACAP-induced long-lasting protection, neurons were transfected with vectors expressing either β-globin (control) or ICER1 plus a peGFP transfection marker. At 24 h post-transfection, neurons were placed in trophically-deprived medium and treated ± PACAP. After a further 24 h, images were taken of GFP-expressing neurons, prior to the transfer of the neurons to PACAP-free, TTX-containing medium to block AP firing. The fate of the transfected neurons was followed at 24 h and 48 h after TTX treatment (see Fig. 3b, schematic). We found that the transfection procedure caused slightly higher rates of neuronal death than in untransfected cells. However, in control-transfected neurons, PACAP treatment promoted significant protection both 24 h and 48 h after the removal of PACAP (Fig. 3b). Importantly, PACAP treatment was not significantly neuroprotective in ICER1-expressing neurons (Fig. 3b), indicating a role for CRE/CREB-dependent gene expression in PACAP-mediated long-lasting neuroprotection.

### AP firing underlies PACAP-induced CREB activation

The fact that PACAP-induced neuroprotection is not observed when neurons are co-treated with TTX suggested that activation of CRE-dependent gene expression could be dependent on AP firing. Indeed, we found this to be the case: TTX treatment alone had little effect on basal activity of a CRE- reporter, but inhibited PACAP-mediated activation by around 80% (Fig. 3c). PKA inhibition by H-89 treatment completely blocked the induction of the CRE reporter by PACAP, *including* the small TTX-insensitive component. Taken together, these data show that direct signaling by PKA is able to support weak activation of CRE-dependent gene expression, but that AP firing is needed for strong CRE-induction and resultant neuroprotection.

To further confirm the role of PKA in both activity-dependent and -independent activation of CREB by PACAP, we studied activation of a CRE reporter in neurons cultured from a mouse deficient in the RIIβ subunit of PKA. In the RIIβ−/− mouse, levels of cAMP-inducible PKA activity within the cortex are lower than wild-type, while basal PKA activity is similar (Brandon *et al.* 1998). We found that both TTX-sensitive and -insensitive components of PACAP-induced CRE-mediated gene expression were lower in RIIβ-null neurons (Fig. 3d). The level of reduction in PACAP-induced CRE-activation in the RIIβ-null neurons was comparable to that seen in the context of the adenylate cyclase activator forskolin (Fig. 3d), confirming that PKA is central to both AP-dependent and -independent components of CREB activation by PACAP.

Given that activity-dependent Ca^2+^ influx can activate Ca^2+^-dependent adenylate cyclases, it was theoretically possible that PKA could play a role in CREB activation *downstream* of AP firing. However, it has been shown that strong firing activity does not cause global levels of cAMP to rise sufficiently high to support PKA signaling to CREB in the nucleus (Pokorska *et al.* 2003). Nevertheless, to investigate this directly we studied the activation of CRE-mediated gene expression by AP firing induced via a PKA-independent mechanism: network disinhibition by the GABA_A_ receptor blocker bicuculline plus the K^+^ channel blocker 4-aminopyridine [to enhance burst frequency (Hardingham *et al.* 2001)]. Induction of CRE-mediated gene expression by bicuculline/4-AP-induced AP firing was *not* lower in RIIβ-null neurons (Fig. 3d). This indicates that cAMP/PKA signaling is not a major mediator of CRE-dependent gene expression *downstream* of AP firing, in agreement with previous studies (Pokorska *et al.* 2003). Collectively these observations support a model whereby PACAP-induced PKA signaling weakly activates CREB directly, but triggers strong CREB activation by promoting AP firing which in turn activates CREB via PKA-independent pathways.

### PACAP-induced CREB phosphorylation does not require AP firing

**Fig. 4:**
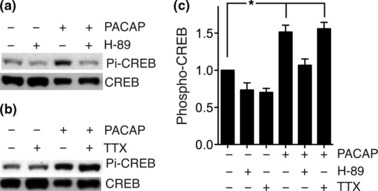
PACAP-mediated induction of serine-133 CREB phosphorylation does not require AP firing. (a–c) PACAP induces phosphorylation of CREB at serine-133 in a TTX-insensitive, PKA-dependent manner. Neurons were pre-treated with TTX or H-89 and then treated for 15 min with PACAP. Protein was harvested and subject to western analysis for phospho-CREB (see Methods, normalized in all cases to total CREB, *p < 0.05, *n* = 4, example blots are shown). (a) and (b) show example westerns and (c) shows quantitation of phospho-CREB levels (normalized to total CREB).

We next investigated which CRE-activating molecular events triggered by PACAP treatment are actually reliant on activity-dependent Ca^2+^ signals, and whether any can be triggered in an activity-independent manner by direct PKA signaling. CREB phosphorylation on serine-133 is essential for CREB activation since it triggers the recruitment of the co-activator CREB binding protein (CBP; Chrivia *et al.* 1993). CREB phosphorylation was induced by PACAP treatment (Fig. 4a). Interestingly, TTX treatment did not interfere with PACAP-induced CREB phosphorylation (Fig. 4b and c), while the PKA inhibitor H-89 blocked CREB phosphorylation (Fig. 4a and c). Serine-133 of CREB is a good substrate for PKA (Gonzalez and Montminy 1989), and these data indicate that PACAP-induced PKA activity is sufficient to result in the direct phosphorylation of CREB, and that AP firing is *not* needed for this particular activation step. However, while CREB serine-133 phosphorylation is necessary for full activation of CREB, it is not sufficient. A key secondary activation step involves the co-activator CRTC (CREB-regulated transcription co-activator), which is subject to Ca^2+^-dependent nuclear import, where it binds to CREB and enhances its affinity for both CBP and the basal transcriptional machinery (Conkright *et al.* 2003; Screaton *et al.* 2004; Zhou *et al.* 2006; Kovacs *et al.* 2007; Li *et al.* 2009).

### PACAP-induced AP firing mediates calcineurin-dependent CRTC1 nuclear import

**Fig. 5:**
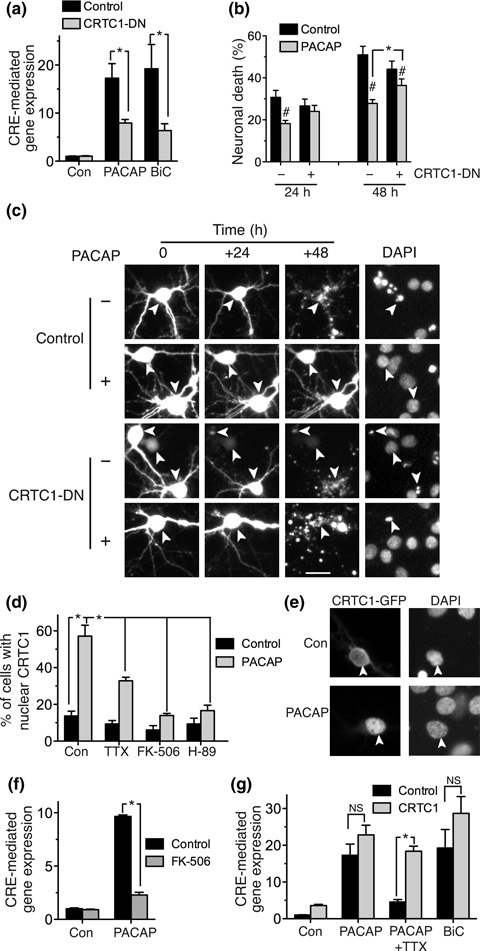
PACAP induces nuclear translocation of CRTC1, necessary for the AP firing-dependent component of CREB activation. (a) CRTC1 dominant negative inhibits PACAP mediated activation of CREB. Neurons were transfected with CRE-luciferase, pTK-Renilla and vectors encoding either a CRTC1 dominant negative mutant or control (β-globin). Neurons were stimulated PACAP or bicuculline plus 4-AP (BiC) (*p < 0.05, *n* = 4). **(**b,c) PACAP mediated long-lasting neuroprotection depends on CRTC1. The experimental protocol is the same as that illustrated schematically in Fig. 3(b). Briefly, neurons expressing GFP plus either CRTC1-DN (dominant negative) or β-globin control were treated ± PACAP 24 h post-transfection and then all cells were placed in TTX-containing medium after a further 24 h, at which point images of GFP-expressing neurons were taken. The fate of these cells was then monitored at 24 and 48 h after this medium change (*p < 0.05, paired *T*-test, *n* = 3; #*p* < 0.05, paired *T*-test comparing control to PACAP within each condition/timepoint). (c) shows example pictures. Scale bar = 20 μm. (d,e) PACAP induces CRTC1 nuclear translocation via activity-dependent calcineurin signaling. Neurons were transfected with a vector encoding GFP-tagged CRTC1. At 24 h post-transfection, neurons were treated with 20 ng/mL leptomycin B for 30 min to block nuclear export [to enable import to be observed more clearly (Kovacs *et al.* 2007)], plus the indicated inhibitors (1 μM TTX, 10 μM H-89 or 10 μM FK-506) and then PACAP added for 30 min prior to fixing of the cells and analysing localisation of GFP-CRTC1 in 400–800 cells per treatment (*p < 0.05, *n* = 4–8). (e) shows example pictures. (f) PACAP-induced activation of CRE-mediated gene expression requires the Ca^2+^-dependent phosphatase calcineurin. Where used, FK-506 was added 1 h prior to PACAP stimulation (*p < 0.05, *n* = 4). (g) CRTC1 over-expression rescues the inhibition of PACAP-mediated CRE activation by TTX. Neurons were transfected with CRE-luciferase, pTK-Renilla and either vectors encoding CRTC1 or β-globin control. 24 h post-transfection the neurons were stimulated with PACAP ± TTX or bicuculline + 4-AP (BiC) as indicated. Over-expression of CRTC1 does not further enhance CRE activation by BiC or PACAP, suggesting that levels are not limiting, however, it strongly enhances levels induced by PACAP in the presence of TTX (*p < 0.05, *n* = 4).

We confirmed the importance of CRTC for CRE activation: expression of a dominant negative mutant of CRTC1 (CRTC1-DN; Zhou *et al.* 2006) strongly inhibited PACAP-induction of the CRE-mediated gene expression, as well as that induced by bicuculline/4-AP treatment (Fig. 5a). We also investigated the importance of CRTC signaling in PACAP-mediated long-lasting neuroprotection, using an identical protocol to that used in Fig. 3(b), except that the ICER-encoding vector was replaced with that of CRTC1-DN. At the 48 h timepoint (after removal of PACAP from the medium), a very small, but statistically significant, amount of PACAP-dependent neuroprotection was still observed at 48 h in CRTC1-DN-expressing neurons. However, levels of neuronal death in CRTC1-DN-expressing neurons previously exposed to PACAP were significantly higher than control-transfected cells previously exposed to PACAP (Fig. 5b and c). Thus, CRTC1-DN interferes with neuroprotection evoked by transient exposure to PACAP, consistent with the role of CREB in this process, and the importance of CRTCs in CREB-mediated gene expression.

Nuclear translocation of CRTC is an important step in the full activation of CREB-dependent gene expression (Screaton *et al.* 2004). We found that PACAP treatment caused the nuclear translocation of CRTC1 that was inhibited by TTX (Fig. 5d and e). Activity-dependent Ca^2+^ influx is known to induce CRTC1 translocation through activation of the Ca^2+^-dependent phosphatase calcineurin. Calcineurin subsequently dephosphorylates CRTC, triggering its nuclear import and co-activation of CREB (Li *et al.* 2009). This mechanism is employed in the case of PACAP signaling: inhibition of calcineurin activity by treatment with the inhibitor FK-506 inhibited PACAP-mediated CRTC1 translocation (Fig. 5d) and PACAP-mediated induction of CRE-mediated gene expression (Fig. 5f). As a control, we wanted to confirm that FK-506 was not inhibiting the induction of activity-dependent Ca^2+^ transients. Using the same methodology as for Fig. 1(d), we found that TTX-sensitive Ca^2+^ elevation in PACAP + FK-506-treated neurons was 100 ± 6.4% of that in neurons treated with PACAP alone (*n* = 3, 25–30 cells analysed per treatment). Thus, the inhibitory effect of FK-506 on PACAP-mediated CRTC1 translocation is not because of any indirect interference in the induction of activity-dependent Ca^2+^ transients.

Taken together, these observations suggest that a key reason why TTX inhibits PACAP-activation of CRE-mediated gene expression is that blockade of CRTC nuclear import renders nuclear levels of CRTC too low to efficiently co-activate CREB. We postulated that if CRTC was indeed limiting, then if we over-expressed CRTC1, then this might rescue the inhibitory effect of TTX on PACAP activation of the CRE reporter. Although over-expressed CRTC1 would be mainly cytoplasmic, we reasoned that since a proportion of it is nuclear then this could rescue the deficiency in nuclear levels. We found this to be the case: over-expression of CRTC1 reversed the inhibitory effect of TTX on PACAP-induction of CREB-dependent gene expression (Fig. 5g). CRTC1 over-expression, however, did not further enhance PACAP activation of CREB-mediated gene expression in the absence of TTX (Fig. 5g), indicating that PACAP-induced firing causes sufficient CRTC nuclear import such that levels of nuclear CRTC are not limiting for efficient co-activation of CREB. Thus, while PACAP activation of direct PKA signaling is sufficient to induce CREB phosphorylation, this is insufficient to activate CREB on its own. Enhancement of AP firing is critical in order to induce calcineurin-dependent CRTC nuclear translocation, an important step in CREB activation and consequent neuroprotection.

## Discussion

**Fig. 6:**
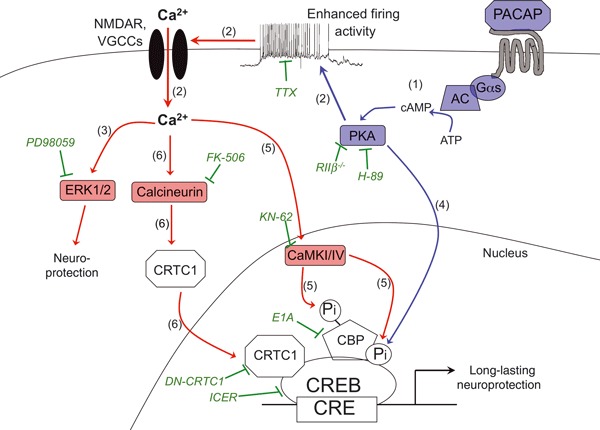
Schematic illustration of the role of activity-dependent Ca^2+^ signaling in PACAP-mediated neuroprotection. Activation of PACAP receptors leads to activation of PKA via the classical G-protein-adenylate cyclase (AC)-cAMP: pathway (1). PKA activation causes an increase in synaptic strength and/or neuronal excitability leading to a strong increase in levels of action potential firing which in turn triggers intracellular Ca^2+^ influx, likely through synaptic receptors (e.g. NMDA receptors) or voltage-gated Ca^2+^ channels (VGCCs): pathway (2). Activation of long-lasting neuroprotection by PACAP requires induction of gene expression mediated by the transcription factor CREB. CREB phosphorylation on serine-133 can be triggered directly by PKA in an AP firing-independent manner: pathway (3). However, this is insufficient to fully activate CREB-mediated gene expression. A key Ca^2+^/activity-dependent pathway involves CRTC1 nuclear translocation through activation of the Ca^2+^-dependent phosphatase calcineurin: pathway (4). Blue arrows and molecules indicate AP firing activity-independent events, while red arrows and molecules highlight the events dependent on AP firing. The pharmacological and genetic inhibitors of the various pathways used in this study are shown in green.

This study shows that certain PACAP-mediated anti-apoptotic signals in cortical neurons are not mediated by direct cAMP/PKA-dependent activation. Instead, the primary role of cAMP/PKA signaling is to enhance neuronal network activity. The resulting AP-dependent Ca^2+^ transients are the direct activators of neuroprotection, and induce a long-lasting phase of protection dependent on activation of CREB-mediated gene expression. These events are illustrated schematically in Fig. 6.

### Modulation of neuronal electrical activity by PACAP and other AC-coupled ligands

The ability of PACAP to induce AP firing in networks of cortical neurons is consistent with the known influence of intracellular cAMP on neuronal excitability. Neurotransmitters, neuropeptides and pharmacological compounds that activate AC are well-known to modulate neuronal excitability, ion channel conductance, and synaptic transmission and plasticity, predominantly through PKA activation (Nguyen and Woo 2003). At the synapse, pharmacological activators of AC, and agonists of AC-coupled receptors such as the D_1_/D_5_ dopamine receptor, or the β-adrenergic receptor, all mimic long term potentiation (LTP) and/or enhance excitatory post-synaptic potentials (Nguyen and Woo 2003). Mice deficient in AC1 and AC8 show deficits in LTP and spatial memory (Nguyen and Woo 2003; Ferguson and Storm 2004). At the molecular level, PKA-mediated GluR1 phosphorylation at serine-845 increases AMPA receptor open probability and stabilizes synaptic location of AMPA receptors trafficked to the synapse during LTP (Banke *et al.* 2000; Esteban *et al.* 2003; Lee *et al.* 2003). PACAP at low doses is known to enhance AMPAR currents via PKA activation (Costa *et al.* 2009) as well as synaptic NMDAR currents (MacDonald *et al.* 2007). Moreover, mice deficient in PACAP have defective LTP at the mossy fibre synapse, implicating endogenous PACAP signaling in synaptic potentiation (Otto *et al.* 2001). In addition to modifying the properties of synaptic glutamate receptors, AC-coupled PKA signaling also can modulate neuronal excitability by controlling the IsAHP. IsAHP is mediated by a Ca^2+^ activated potassium current which is activated in response to bursts of AP firing. This is a key negative regulator of neuronal excitability, inducing a prolonged state of hyperpolarization, and this is in turn negatively regulated by AC-coupled PKA activity induced either pharmacologically (e.g. forskolin) or by treatment with AC-coupled ligands (e.g. dopamine) (Pedarzani and Storm 1995; Lancaster *et al.* 2006; Hu *et al.* 2011). PACAP treatment itself leads to inhibition of IsAHP in cortical pyramidal neurons (Hu *et al.* 2011), which could contribute to the enhanced AP firing that we observe (Fig. 1).

### PACAP-induced AP firing promotes CREB-dependent neuroprotection

The CREB family of transcription factors is known to be an important mediator of activity-dependent gene expression (Lonze and Ginty 2002). The potential of CREB family-regulated gene products to promote neuronal survival was first demonstrated in the context of neurotrophin signaling (Bonni *et al.* 1999; Riccio *et al.* 1999) and exogenous over-expression (Walton *et al.* 1999). In addition, studies of mice where CREB and/or CREB family members have been deleted also point to a pro-survival role for CREB *in vivo* both pre- and postnatally (Lonze *et al.* 2002; Mantamadiotis *et al.* 2002). CREB-dependent gene expression is causally linked to the long-lasting phase of activity-dependent neuroprotection against apoptotic insults (Papadia *et al.* 2005) and data presented in this study supports the idea that PACAP treatment recruits this activity-dependent neuroprotective pathway.

Activation of CREB-mediated gene expression requires serine-133 phosphorylation which is necessary to recruit CBP, a transcriptional cofactor, to CREB (Chrivia *et al.* 1993). Several Ca^2+^-activated kinase cascades can mediate this event, including the Ras-extracellular signal regulated kinase 1/2 pathway and also nuclear Ca^2+^/calmodulin-dependent protein kinase (CaM kinase) (Soriano and Hardingham 2007). However, PACAP induced CREB phosphorylation does not require these activity-dependent pathways, since even when AP firing is blocked CREB phosphorylation is still observed (Fig. 4). This is consistent with the fact that PKA is also a CREB kinase and indicates that PACAP-induced PKA activity is strong enough to mediate this event directly.

However, direct PKA activity induced by PACAP is not sufficient to induce subsequent activation steps, including nuclear translocation of CRTCs. CRTCs enhance the interaction of CREB with the TAF(II)130 component of TFIID following its recruitment to the promoter (Conkright *et al.* 2003). Calcineurin promotes nuclear translocation of CRTC2 through calcineurin-mediated dephosphorylation of serine-171 (Screaton *et al.* 2004). Translocation can be enhanced/synergized by PKA signaling with causes the inhibition of the serine-171-kinase – salt-inducible kinase-2 (Screaton *et al.* 2004). CRTC1 is the major isoform in the brain and is a key regulator of CREB-dependent gene expression (Kovacs *et al.* 2007; Li *et al.* 2009). Ca^2+^ signals promote the nuclear translocation of CRTC1, dependent on calcineurin signaling which directly dephosphorylates CRTC1 (Bittinger *et al.* 2004). Analogously with CRTC2, cAMP signals can also trigger the translocation of CRTC1 (Bittinger *et al.* 2004), most likely through the inhibition of salt-inducible kinase-mediated phosphorylation. In neurons, calcineurin activation is sufficient to trigger CRTC1 translocation (Li *et al.* 2009). The requirement for AP firing and calcineurin signaling for PACAP treatment to induce CRTC1 translocation strongly indicates that PACAP-induced PKA activity is not strong enough on its own to promote sufficient CRTC1 translocation directly, although may be playing a supporting role.

Another more recently discovered role for CRTC is in assisting the recruitment of CREB’s co-activator CBP to phospho(serine-133) CREB (Ravnskjaer *et al.* 2007). We and others have shown previously that CBP itself is activated by Ca^2+^ influx in neurons (via CaM kinase-dependent phosphorylation) which contributes to activation of CRE-mediated gene expression (Chawla *et al.* 1998; Hardingham *et al.* 1999; Impey *et al.* 2002). Thus, CBP activation may also contribute to the activation of CRE-mediated gene expression by PACAP-induced firing activity. Indeed, we observe strong activity-dependent activation of CBP’s transactivating potential by PACAP, which is both dependent on firing activity and CaM kinase activity (Baxter and Hardingham, unpublished observations).

### PACAP prevents neuronal loss and dysfunction *in vivo*: potential role of enhanced AP firing

PACAP has been reported to protect neurons against a variety of insults including ceramide, glutamate and hydrogen peroxide-induced death (Vaudry *et al.* 2009), insults that synaptic activity also protects against (Lee *et al.* 2005; Papadia *et al.* 2005, 2008). Importantly, activation of CREB-mediated gene expression is implicated in activity-dependent protection against both apoptotic and excitotoxic insults (Lee *et al.* 2005; Papadia *et al.* 2005). Based on this study, it may be that indirect activity-dependent signaling to CREB contributes to the neuroprotective effects of PACAP *in vitro* and also begs the question as to whether any of its *in vivo* effects are similarly because of enhancing neuronal activity.

*In vivo* administration of PACAP reduces neuronal loss in the substantia nigra in acute models of Parkinson’s Disease: 6-Hydroxydopamine and MPTP treatment (Reglodi *et al.* 2004, 2006; Wang *et al.* 2008). However, most neuroprotective studies on PACAP have centred on excitotoxic trauma: principally stroke, traumatic brain injury (TBI) and retinal injury. PACAP crosses the blood brain barrier and can be administered intravenously to decrease damage in several models of ischemia and is effective even when administered several hours after the ischemic episode (Uchida *et al.* 1996; Reglodi *et al.* 2000; Chen *et al.* 2006; Ohtaki *et al.* 2008). Enhanced neuronal AP firing is known to protect neurons against excitotoxic cell death including ischemic conditions (Lee *et al.* 2005; Tauskela *et al.* 2008) and so the notion that PACAP can reduce neuronal damage in part by promoting AP firing is plausible. PACAP is also highly effective in ameliorating damage to the retina in a variety of trauma models, including excitotoxic and ischemic injury (Atlasz *et al.* 2010). In addition, post-insult PACAP treatment also reduces the extent of axonal damage following TBI (Farkas *et al.* 2004; Tamas *et al.* 2006b). TBI is characterised by brief acute hyperactivity of ionotropic glutamate receptors, including NMDA receptors, which mediate acute excitotoxic damage, followed by sustained loss of function (Biegon *et al.* 2004; Yaka *et al.* 2007). As such the NMDA receptor has been proposed to rapidly switch between ‘destructive’ and ‘recovery’ roles (Biegon *et al.* 2004; Yaka *et al.* 2007). In the immature brain, treatment with NMDAR antagonists reduces primary excitotoxic death but exacerbates secondary apoptosis, resulting in increased overall death (Pohl *et al.* 1999). By promoting AP firing, PACAP may boost the recovery phase post-injury by mechanisms related to those described in this study, as well as others more specific to the activity-dependent protection of axons.

Of course, enhanced neuronal activity is very unlikely to mediate all the effects of PACAP in the CNS: direct activity-independent effects are likely to be exerted in neurons as well. Moreover, there are well-documented neuroprotective effects of PACAP acting indirectly via non-neuronal cells. For example, PACAP stimulates the astrocytic release of neuroprotective IL-6 (Ohtaki *et al.* 2008) and also suppresses microglial activation, thus reducing the release of potentially harmful cytokines that can form part of the post-ischemic response (Gonzalez-Rey *et al.* 2007; Vaudry *et al.* 2009). Nevertheless, the impact of PACAP on neuronal activity should be taken into account when assessing the mechanism and extent of any therapeutic effect.

## Abbreviations used

AMPA: α-amino-3-hydroxy-5-methylisoxazole-4-propionate
AP: action potential
CaM kinase: Ca^2+^/calmodulin-dependent protein kinase
CBP: CREB binding protein
CRE: cAMP response element
CREB: CRE binding protein
CRTC: CREB-regulated transcription co-activator
CRTC1-DN: dominant negative CRTC1
GFP: green fluorescent protein
ICER: inducible cAMP early repressor
IsAHP: Apamin-insensitive slow after-hyperpolarization current
LTP: long term potentiation
PACAP: pituitary adenylate cyclase-activating peptide
PKA: protein kinase A
TBI: traumatic brain injury
TTX: tetrodotoxin
TORC1: transducer of regulated CREB activity 1
